# INDICATORS OF WELLBEING AND SELF- AND INFORMANT-REPORTED COGNITIVE DECLINE IN OLDER ADULTS

**DOI:** 10.1093/geroni/igad104.3431

**Published:** 2023-12-21

**Authors:** Karysa Britton, Patrick Hill, Ryan Bogdan, Thomas Oltmanns, Emily Willroth

**Affiliations:** Washington University in St. Louis, St. Louis, Missouri, United States; Washington University in St. Louis, St. Louis, Missouri, United States; Washington University in St. Louis, St. Louis, Missouri, United States; Washington University in St. Louis, St. Louis, Missouri, United States; Washington University in St. Louis, St. Louis, Missouri, United States

## Abstract

Wellbeing has been associated with objective cognitive decline and is a promising target for dementia prevention. Less is known about associations between wellbeing and subjective cognitive decline (SCD), an early indicator for progression to Alzheimer’s Disease and related dementias (ADRD). Given the importance of early detection and intervention in ADRD, the current study assessed whether positive and negative indicators of wellbeing were associated with SCD via self- and informant-reports using zero-inflated Poisson regressions. A total of 1,014 participants (MAge=71.8, SD=3.1) were included in analyses. The sample was representative of both males (55%) and females (45%) and was racially diverse (65% White, 32% Black/African American, 2% other races). SCD was assessed with the AD8 Dementia Screening Interview (self- and informant-report versions), sense of purpose with the Life Engagement Test, life satisfaction with the Satisfaction with Life Scale, depressive symptoms with the Beck Depression Inventory-II, and loneliness with the UCLA Loneliness scale. Higher positive indicators of wellbeing were associated with lower SCD via self- and informant-reports. Higher levels of depression were associated with greater SCD via self-report. Higher levels of life satisfaction attenuated the association between depressive symptoms and SCD via self-report. These associations were statistically significant (p < .05) and held after applying False Discovery Rate correction. Effect sizes ranged from small to medium (ORPositive Wellbeing Indicators = 0.84-0.89, ORDepression = 1.34). These findings extend past wellbeing and objective cognitive decline research by demonstrating that positive and negative indicators of wellbeing are also associated with SCD assessed via self- and informant-reports.

